# Microbiome dataset of eukaryotic and fungal communities in the bulk soil and root of wild *Brassica napus* in South Korea

**DOI:** 10.1016/j.dib.2022.108457

**Published:** 2022-07-11

**Authors:** Seong-Jun Chun

**Affiliations:** LMO Team, National Institute of Ecology, 1210 Geumgang-ro, Maseo-myeon, Seocheon 33657, Republic of Korea

**Keywords:** Brassica napus, Eukaryotic community, Fungal community

## Abstract

This article describes the dataset of the eukaryotic and fungal microbiome in bulk soil and root of wild *Brassica napus* at five different grassland sites in South Korea. The microbiome datasets were obtained using Illumina MiSeq sequencing of the 18S rRNA gene and ITS1 gene. The raw sequences and metadata used for analysis are available at the National Center for Biotechnology Information (NCBI) (BioProject ID: PRJNA821335). Raw data were clustered into amplicon sequence variants (ASVs) using the DADA2 pipeline and aligned against the SILVA 132 reference database and UNITE database. A total of 5702 eukaryotic ASVs (1,913,372 reads) and 4565 fungal ASVs (9,032,969 reads) were extracted after quality-filtering. Rhizaria was the most dominant eukaryote at the class level, and Olpidiomycetes was the dominant fungal class in this dataset. As unintended releases of transgenic *B. napus* have been reported in South Korea [1], the microbiome datasets produced in this work will be used as the foundation for environmental risk assessment to understand the potential effect of released transgenic *B. napus* on the natural ecosystem.

## Specifications Table

**Table:** 

Subject	Environmental Genomics and Metagenomics
Specific subject area	ITS and 18S Metagenomics of wild *Brassica napus*
Type of data	Amplicon sequencing data of ITS and 18S rRNA region
How the data were acquired	DNA sequences: Illumina Miseq platform Data processing: DADA2 v. 2019.1. Data analysis: R v. 3.6.1.
Data format	Raw, filtered, and analyzed
Description of data collection	Root and bulk soil of wild *Brassica napus* were collected and used for DNA library construction based on amplicon sequencing of the 18s rRNA and Internal Transcribed Spacer (ITS) regions.
Data source location	Institution: National Institute of Ecology Plant: *Brassica napus* Region (Latitude and longitude): Buyeo (36° 9′12.21"N, 127° 0′0.79"E); Gurye (35°13′41.69"N, 127°27′14.48"E); Naju (35°00′3.16″N, 126°42′7.58″E); Sangju (36°26′21.53"N, 128°15′32.85"E); and Seosan (36°42′35.04"N, 126°32′36.90"E) Country: South Korea Month and years: April, 2021
Data accessibility	Raw sequences Repository name: NCBI SRA Data identification number: PRJNA821335 Direct URL to data: https://www.ncbi.nlm.nih.gov/bioproject/821335 Accessions: SAMN27065993-SAMN27066002 ASV tables Repository name: Mendeley Data Data identification number: doi: 10.17632/kvnj4kxvbr.1 Direct URL to data: http://dx.doi.org/10.17632/kvnj4kxvbr.1

## Value of the Data

• These eukaryotic and fungal microbiome datasets can be used for understanding microbial dynamics in the rhizosphere of wild *Brassica napus* grown in the natural ecosystem.
• These data are valuable for understanding the co-occurrence patterns and interactions among eukaryotes and fungus in the rhizosphere.
• Crop and environmental scientists can use the datasets for potential environmental risk assessments of transgenic *B. napus*.

## 1 Data Description

**Fig. 1:**
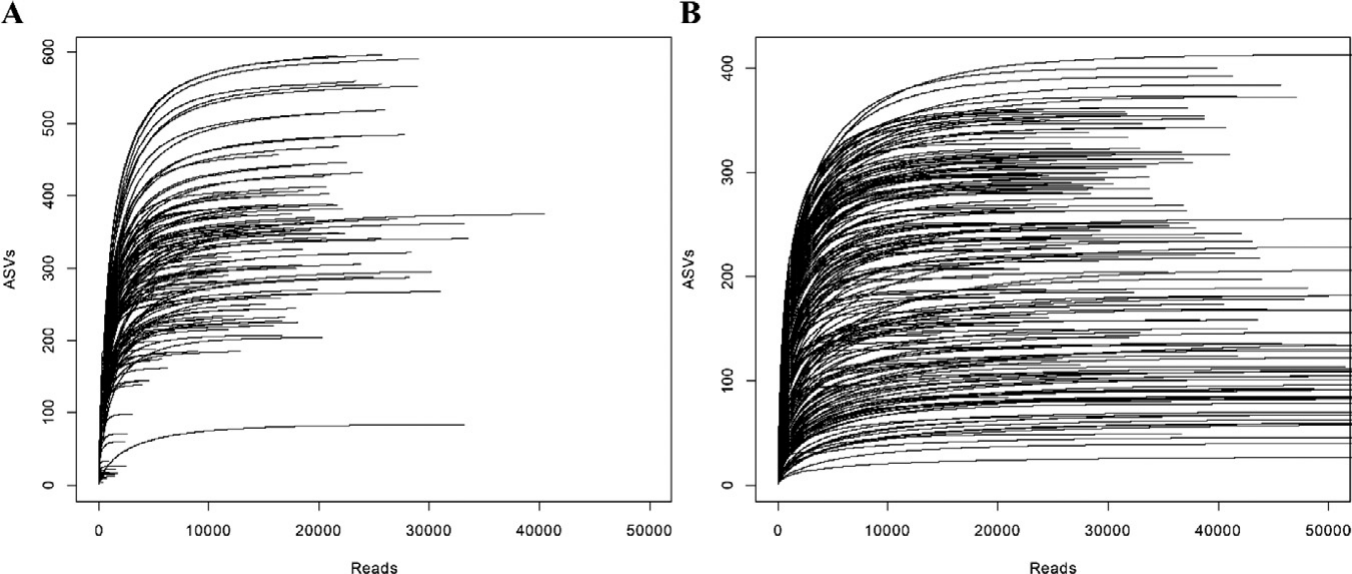
Rarefaction curves. (A) Eukaryotic samples and (B) fungal samples.

**Fig. 2:**
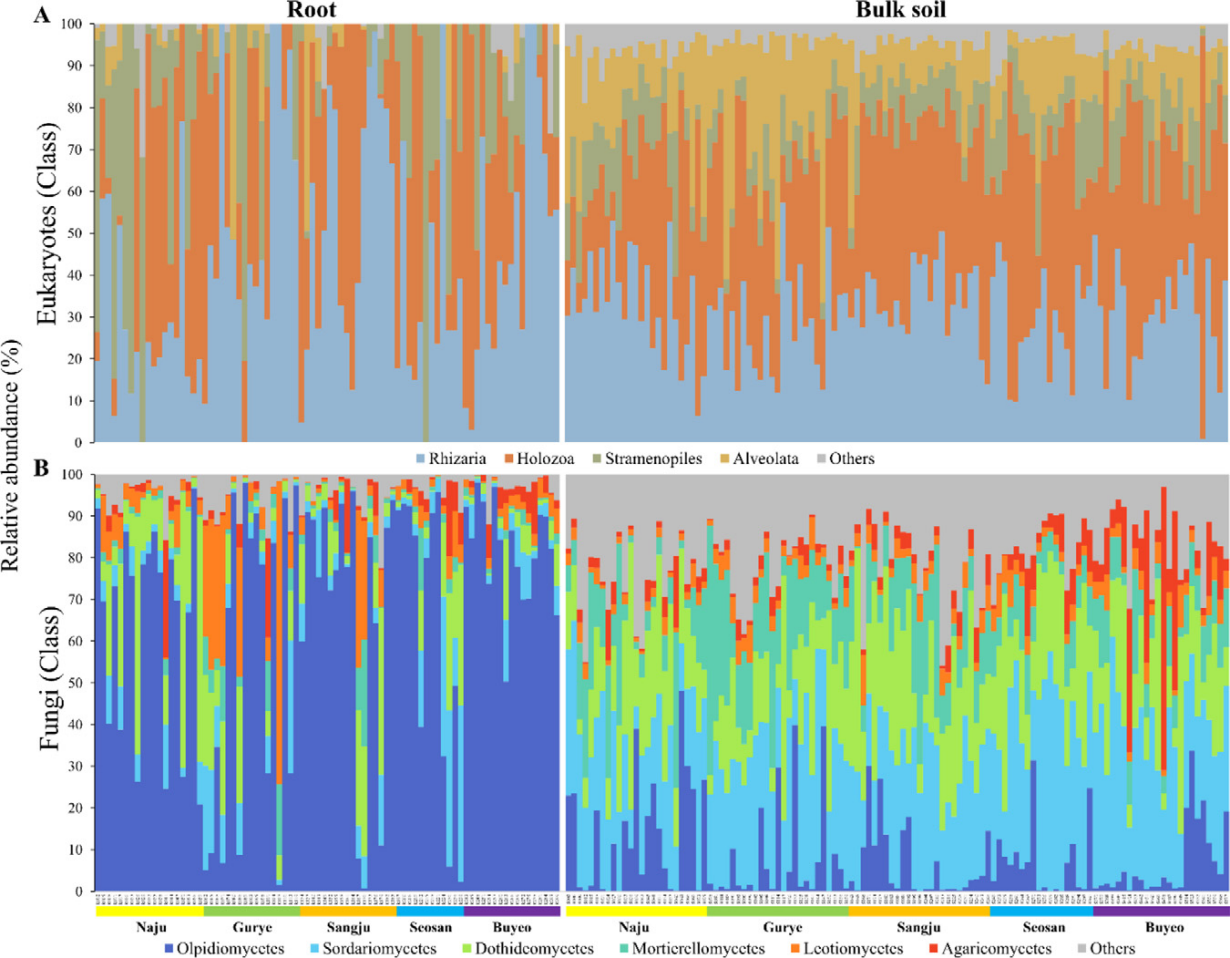
The relative abundance of eukaryotic and fungal communities in bulk soil and root samples at the class level.

The data in this dataset describe the taxonomic profiles of bulk soil and root samples of wild *Brassica napus* from five different grassland sites in South Korea. A total of 199 samples were collected from the bulk soil and root of *B. napus*. Amplicon libraries were constructed for eukaryotic and fungal communities by MiSeq sequencing. A total of 5702 eukaryotic amplicon sequence variants (ASVs; 1,913,372 reads) and 4565 fungal ASVs (9,032,969 reads) were extracted after quality- and chimera-filtering, as described in the Material and Methods section. The raw pair-end FASTQ and metadata files are deposited in the NCBI SRA database under the BioProject ID PRJNA821335 (.fastq format). Metadata file provides the following information about samples: primer set, isolation source, date of sample collection, sampling sites, and technical batch of sequencing. Processed ASV tables and taxonomic assignments are available at Mendeley Data with the DOI shown in the Specifications table. The rarefaction curves of each sample are shown in Fig. 1, which supported the depth of sequencing for further analysis. Fig. 2 displays the relative abundance of eukaryotic and fungal communities at the class level. Rhizaria (36.7% ± 21.7%) was the most dominant class (Fig. 2A), followed by Holozoa (35.2% ± 23.2%) and Stramenopiles (13.8% ± 16.3%). The relative abundance of Alveolata was approximately five times higher in bulk soil samples than in root samples. At the ASV level, the most abundant eukaryotic ASV was assigned to the order *Haplotaxida* (Table 1). For fungal community, Olpidiomycetes (32.3% ± 35.5%), Sordariomycetes (19.7% ± 14.5%), and Dothideomycetes (16.3% ± 14.1%) were the dominant groups (Fig. 2B). The relative abundance of major eukaryotic and fungal ASVs are given in Table 1. At the ASV level, the most abundant eukaryotic and fungal ASV were assigned to the genus *Haplotaxida* and *Olpidium* (Table 1).

**Table 1:** The relative abundance and taxonomy of major eukaryotic and fungal amplicon sequence variants.

ASVs	Root (%)	Bulk soil (%)	Taxonomy
ASV_F0001	58.5	7	Fungi; Olpidiomycota; Olpidiomycetes; Olpidiales; Olpidiaceae; *Olpidium*
ASV_F0002	1.7	3.2	Fungi; Ascomycota; Dothideomycetes; Capnodiales; Cladosporiaceae; *Cladosporium*
ASV_F0003	0.5	4.2	Fungi; Ascomycota; Sordariomycetes; Hypocreales; Nectriaceae; *Fusarium*
ASV_F0004	0.6	4	Fungi; Mortierellomycota; Mortierellomycetes; Mortierellales; Mortierellaceae; *Mortierella*
ASV_F0005	1.1	2.6	Fungi; Ascomycota; Dothideomycetes; Pleosporales; Pleosporaceae; *Alternaria*
ASV_F0006	1.6	2	Fungi; Ascomycota; Dothideomycetes; Pleosporales; Didymellaceae; *Epicoccum*
ASV_F0007	1.9	0.2	Fungi; Olpidiomycota; Olpidiomycetes; Olpidiales; Olpidiaceae; *Olpidium*
ASV_F0008	0.7	2.1	Fungi; Mortierellomycota; Mortierellomycetes; Mortierellales; Mortierellaceae; *Mortierella*
ASV_F0009	1.9	0.7	Fungi; Olpidiomycota; Olpidiomycetes; Olpidiales; Olpidiaceae; *Olpidium*
ASV_F0010	2	0.5	Fungi; Ascomycota; Leotiomycetes; Helotiales; NA; NA
ASV_E0001	2	3.6	Eukaryota; Opisthokonta; Holozoa; Metazoa (Animalia); Oligochaeta; *Haplotaxida*
ASV_E0002	1.6	3	Eukaryota; Opisthokonta; Holozoa; Metazoa (Animalia); Oligochaeta; *Haplotaxida*
ASV_E0003	0.2	2.6	Eukaryota; Opisthokonta; Holozoa; Metazoa (Animalia); Arachnida; *Acari*
ASV_E0004	7.6	2.3	Eukaryota; Opisthokonta; Holozoa; Metazoa (Animalia); Chromadorea; *Rhabditida*
ASV_E0005	0.3	2.1	Eukaryota; SAR; Alveolata; Apicomplexa; Eugregarinorida; *Gregarina*
ASV_E0006	0.4	2	Eukaryota; SAR; Rhizaria; Cercozoa; Thecofilosea; NA
ASV_E0007	0	0.8	Eukaryota; Opisthokonta; Holozoa; Metazoa_(Animalia); Oligochaeta; *Haplotaxida*
ASV_E0008	0.9	1	Eukaryota; Opisthokonta; Holozoa; Metazoa_(Animalia); Oligochaeta; *Haplotaxida*
ASV_E0009	0.1	1.3	Eukaryota; Opisthokonta; Holozoa; Metazoa_(Animalia); Enoplia; *Triplonchida*
ASV_E0010	0	0.5	Eukaryota; Opisthokonta; Holozoa; Metazoa_(Animalia); Oligochaeta; *Haplotaxida*

## 2 Experimental Design, Materials and Methods

### 2.1 Study Site and Sampling Design

A total of 82 bulk soil and 117 root samples were collected between April 14 and April 30, 2021, from the following five sites: Buyeo (36° 9ʹ12.21ʺ N, 127° 0ʹ0.79ʺ E), Gurye (35° 13ʹ41.69ʺ N, 127° 27ʹ14.48ʺ E), Naju (35° 00ʹ3.16ʺ N, 126° 42ʹ7.58ʺ E), Sangju (36° 26ʹ21.53ʺ N, 128° 15ʹ32.85ʺ E), and Seosan (36° 42ʹ35.04ʺ N, 126° 32ʹ36.90ʺ E). Sampling sites were selected to include the natural habitats of *B. napus* with diverse plant species that had experienced low levels of disturbance by humans. Plants at the flowering stage and of similar size were selected. After digging up each plant with an ethanol-sterilized shovel to minimize root damage, sampling was conducted for bulk soil and root. Bulk soil samples were collected from the soil that fell off the plant following light shaking, and the parts that did not contain plant debris and root were gathered. After collecting the bulk soil samples, the plant was vigorously shaken to remove loosely bound soil, and the roots and tightly bound soil were collected together. The shovel, forceps, and blades were cleaned with 70% ethanol and washed with sterile water between the handling of each sample to minimize contamination. The samples were stored at −80 °C until DNA extraction.

### 2.2 DNA Extraction and Sequencing

DNA was extracted using DNeasy PowerMax® soil kits (Qiagen, Hilden, Germany) according to the manufacturer's instructions, and the quality and concentration of extracted DNA were evaluated using a NanoDrop 2000 spectrophotometer (Thermo Scientific, DE, USA). The eukaryotic 18S rRNA gene was amplified using a universal primer set with overhang adapter sequences, TAReuk454FWD1/TAReukREV3 (TAReuk454FWD1: 5′-CCAGCASCYGCGGTAATTCC-3′; TAReukREV3: 5′-ACTTTCGTTCTTGATYRA-3′), which targets the V4 region of the 18S rRNA gene [2]. The ITS1 gene was amplified using a universal primer set with overhang adapter sequences, ITS1F_KYO1/ITS2_KYO2 (ITS1F_KYO1: 5′-CTHGGTCATTTAGAGGAASTAA-3′; ITS2_KYO2: 5′-TTYRCTRCGTTCTTCATC-3′) [3]. Dual-PCR amplification, purification, and quantification were performed to prepare Illumina amplicon libraries according to the method described in previous studies [2], [3], [4]. Briefly, PCR assays were conducted in the ProFlex PCR system (Applied Biosystems, CA, USA) using TaKaRa Ex Taq™ Hot Start Version (TaKaRa Bio, Shiga, Japan). The annealing temperatures for PCR were 53 °C and 55 °C for the 18S rRNA gene and ITS1 gene, respectively. PCR products were purified using a 1:1 ratio of AmpureXP bead (Beckman Coulter, IN) and quantified using Quant-iT™ PicoGreen® dsDNA detection kits (Invitrogen, CA, USA). The final products were used for paired-end read sequencing reactions and sequenced using MiSeq (2 × 300 bp reads) obtained from Macrogen Corporation (Seoul, South Korea).

### 2.3 Bioinformatic Analysis

To explore the ASV profiles of eukaryotic and fungal communities, the ASVs of the 18S rRNA gene and ITS gene were calculated using DADA2 (version 1.16), according to the pipeline workflow 1.16 and 1.8 for the 18S rRNA gene and ITS gene, respectively (accessed date: March 2022, https://benjjneb.github.io/dada2/tutorial.html and https://benjjneb.github.io/dada2/ITS_workflow.html) in R [5]. In detail, filtering was performed with the DADA2′s ‘filterAndTrim’ command with the following settings for the 18S rRNA gene data set: truncLen = c(250,220), trimLeft = c(16,17), maxN = 0, maxEE = c(2,2), truncQ = 2, rm.phix = TRUE. Chimeric ASVs were removed with the method ‘consensus’ by using ‘removeBimeraDenovo’ command. The DADA2 formatted Silva database (release 132) was used to align and classify the sequences of the 18S rRNA gene [6]. For the ITS1 gene, filtering was performed with the DADA2′s ‘filterAndTrim’ command with the following settings: minLen = 50, maxN = 0, maxEE = c(2,2), truncQ = 2, rm.phix=TRUE. The UNITE database (UNITE general FASTA release for Fungi 2. Version 10.05.2021.) was used to align and classify the sequences [7]. Subsequently, any reads assigned as chloroplast and fungal sequences were removed in the 18S rRNA dataset and chloroplast sequences in the ITS dataset. ASVs that comprised only singletons, doubletons, and tripletons were not further analyzed. Moreover, ASVs that appeared in at least two samples were used for further analysis. Rarefaction curves were constructed by using ‘rarecurve’ function from the Vegan package [8].

## Ethics Statements

The work did not involve human subjects, animals, cell lines, or endangered species of wild fauna and flora.

## CRediT authorship contribution statement

**Seong-Jun Chun:** Conceptualization, Methodology, Writing – review & editing.

## Declaration of Competing Interest

The author declares that they have no known competing financial interests or personal relationships which have or could be perceived to have influenced the work reported in this article.

## Data Availability

Raw data for ITS and 18S Metagenomics of wild *Brassica napus* (Original data) (Mendeley Data). Eukaryotic and fungal community of wild *Brassica napus* in South Korea (Original data) (NCBI SRA-PRJNA821335).

## References

[1] Kim I.R., Lim H.S., Choi W., Kang D.I., Lee S.Y., Lee J.R. (2020). Monitoring living modified canola using an efficient multiplex PCR assay in natural environments in South Korea. Appl. Sci..

[2] Stoeck T., Bass D., Nebel M., Christen R., Jones M.D., Breiner H.W., Richards T.A. (2010). Multiple marker parallel tag environmental DNA sequencing reveals a highly complex eukaryotic community in marine anoxic water. Mol. Ecol..

[3] Toju H., Tanabe A.S., Yamamoto S., Sato H. (2012). High-coverage ITS primers for the DNA-based identification of ascomycetes and basidiomycetes in environmental samples. PloS ONE.

[4] Bourlat S.J., Haenel Q., Finnman J., Leray M. (2016).

[5] Callahan B.J., McMurdie P.J., Rosen M.J., Han A.W., Johnson A.J.A., Holmes S.P. (2016). DADA2: high-resolution sample inference from illumina amplicon data. Nat. Methods.

[6] Quast C., Pruesse E., Yilmaz P., Gerken J., Schweer T., Yarza P., Peplies J., Glöckner F.O. (2012). The SILVA ribosomal RNA gene database project: improved data processing and web-based tools. Nucleic Acids Res..

[7] Abarenkov K., Zirk A., Piirmann T., Pöhönen R., Ivanov F., Nilsson R.H., Kõljalg U. (2021). UNITE general FASTA release for Fungi 2. Version 10.05.2021. UNITE Community.

[8] J. Oksanen, F. Guillaume Blanchet, M. Friendly, R. Kindt, P. Legendre, D. McGlinn, P.R. Minchin, R.B. O'Hara, G.L. Simpson, P. Solymos, M. Henry H. Stevens, E. Szoecs, H. Wagner (2019). vegan: community ecology package. R package version 2.5-5. https://CRAN.R-project.org/package=vegan.

